# An International Study of the Ability and Cost-Effectiveness of Advertising Methods to Facilitate Study Participant Self-Enrolment Into a Pilot Pharmacovigilance Study During Early Pregnancy

**DOI:** 10.2196/publichealth.5366

**Published:** 2016-03-18

**Authors:** Jonathan Luke Richardson, Sally Stephens, Simon Hugh Lynton Thomas, Anna Jamry-Dziurla, Lolkje de Jong-van den Berg, Priscilla Zetstra - van der Woude, Maja Laursen, Valerie Hliva, Shahrul Mt-Isa, Alison Bourke, Nancy A Dreyer, Stella CF Blackburn

**Affiliations:** ^1^ The UK Teratology Information Service Newcastle upon Tyne Hospitals NHS Foundation Trust Newcastle upon Tyne United Kingdom; ^2^ Institute of Cellular Medicine Newcastle University Newcastle upon Tyne United Kingdom; ^3^ Department of Medical Genetics Poznan University of Medical Sciences Poznan Poland; ^4^ Unit of PharmacoEpidemiology and PharmacoEconomics Department of Pharmacy University of Groningen Groningen Netherlands; ^5^ Department of Data Delivery and Medicinal Product Statistics The Danish Health Data Authority Copenhagen Denmark; ^6^ Quintiles Real-World and Late Phase Research St. Prex Switzerland; ^7^ Imperial Clinical Trials Unit School of Public Health Imperial College London London United Kingdom; ^8^ Real World Evidence Solutions IMS Health London United Kingdom; ^9^ Quintiles Real-World & Late Phase Research Cambridge, MA United States; ^10^ Quintiles Real-World and Late Phase Research Reading United Kingdom

**Keywords:** teratogen, surveillance, pregnancy, pharmacovigilance recruitment, advertisement

## Abstract

**Background:**

Knowledge of the fetal effects of maternal medication use in pregnancy is often inadequate and current pregnancy pharmacovigilance (PV) surveillance methods have important limitations. Patient self-reporting may be able to mitigate some of these limitations, providing an adequately sized study sample can be recruited.

**Objective:**

To compare the ability and cost-effectiveness of several direct-to-participant advertising methods for the recruitment of pregnant participants into a study of self-reported gestational exposures and pregnancy outcomes.

**Methods:**

The Pharmacoepidemiological Research on Outcomes of Therapeutics by a European Consortium (PROTECT) pregnancy study is a non-interventional, prospective pilot study of self-reported medication use and obstetric outcomes provided by a cohort of pregnant women that was conducted in Denmark, the Netherlands, Poland, and the United Kingdom. Direct-to-participant advertisements were provided via websites, emails, leaflets, television, and social media platforms.

**Results:**

Over a 70-week recruitment period direct-to-participant advertisements engaged 43,234 individuals with the study website or telephone system; 4.78% (2065/43,234) of which were successfully enrolled and provided study data. Of these 90.4% (1867/2065) were recruited via paid advertising methods, 23.0% (475/2065) of whom were in the first trimester of pregnancy. The overall costs per active recruited participant were lowest for email (€23.24) and website (€24.41) advertisements and highest for leaflet (€83.14) and television (€100.89). Website adverts were substantially superior in their ability to recruit participants during their first trimester of pregnancy (317/668, 47.5%) in comparison with other advertising methods (*P*<.001). However, we identified international variations in both the cost-effectiveness of the various advertisement methods used and in their ability to recruit participants in early pregnancy.

**Conclusions:**

Recruitment of a pregnant cohort using direct-to-participant advertisement methods is feasible, but the total costs incurred are not insubstantial. Future research is needed to identify advertising strategies capable of recruiting large numbers of demographically representative pregnant women, preferentially in early pregnancy.

## Introduction

### Medication Use in Pregnancy

Maternal medication use during pregnancy is common [1] and is thought to have increased substantially over the last 30 years [2]. However, adequate nonconflicting clinical evidence concerning the fetal effects of maternal medication use in pregnancy takes many years to collect [3]. Safety data is therefore often lacking, particularly for newly marketed medications [3], and this may impact upon both maternal medication compliance [4] and health care professional prescribing [5]. The paucity of data and the potential consequences this may have for both maternal and fetal health, thereby identifies a clear need for improved pharmacovigilance (PV) research in the field. For ethical reasons, and in order to provide sufficient statistical power, this research is predominantly performed using post-marketing pharmacoepidemiological (PE) approaches [6]. However, many of the more commonly used PE methods have been associated with numerous biases and limitations [7] highlighting the need for novel PE approaches to pregnancy PV research.

### Pregnancy Pharmacovigilance

The direct collection of health and medication use data from medicines consumers has proven beneficial in the monitoring of adverse drug reactions [8-10]. Therefore, gestational exposure and pregnancy outcome data collected in a prospective manner directly from pregnant women may provide a useful data source for pregnancy PV research. In 2009, a large collaborative European research program, the Pharmacoepidemiological Research on Outcomes of Therapeutics by a European Consortium (PROTECT) project [11], was funded with the aim of addressing key methodological limitations in PV/PE research. One of the specific aims of the PROTECT project was to explore the feasibility of enhancing the early detection of adverse drug reactions using modern communication methods to collect PV surveillance data. Through employing modern communication techniques in pregnancy PV research, such as website reporting, it may be possible to collect data from a large number of study participants in a manner, which is less researcher time intensive than some traditional epidemiological methods [12]. As data are collected directly from the patient, it may also be reasonable to expect fewer misclassification errors, particularly for socially sensitive details, such as use of alcohol, tobacco, or illicit drugs, or details regarding over-the-counter medication, which are not easily collected from population-based registers of health care data. More specifically for pregnancy PV studies, as self-reporting does not require notification of pregnancy to a health professional, data reported directly from a patient could be collected earlier in pregnancy; hence, providing surveillance over a time period not easily covered by some of the common PV/PE surveillance techniques.

### Study Objectives

Given these potential advantages, the PROTECT pregnancy study was designed to explore whether pregnant women could be recruited to self-provide detailed information to an automated data collection tool, thereby enabling the prospective collection of gestational medication use, lifestyle details, and pregnancy outcomes. Here, we describe recruitment achieved by the direct-to-participant advertisement methods used to recruit participants to this study and compare their cost-effectiveness and the stage of pregnancy at which these methods recruited participants to the study.

## Methods

### PROTECT Pregnancy Study

The PROTECT pregnancy study is a prospective, non-interventional descriptive pilot-study of self-reported medication use and obstetric outcomes as provided by a cohort of pregnant women. The research protocol was created by a collaboration of public and private institutions, with researchers from public health authorities and academic institutions leading the research in each of the four study locations: Denmark, the Netherlands, Poland, and the United Kingdom. Ethical review of the study was required in Poland (Ethics Committee of the Poznań University of Medical Sciences) and the United Kingdom (National Research Ethics Service Committee North East - Sunderland). In the Netherlands as the data being collected were considered anonymous the study was granted an ethical review waiver from a regional research assessment board (Regionale Toetsingscommissie Patiëntgebonden Onderzoek, Leeuwarden), while in Denmark non-interventional epidemiological surveys are considered exempt from the requirement for ethical review [13].

### Study Participants

All study participants were required to provide informed consent for participation. In Denmark, the Netherlands, and the United Kingdom informed consent to participate was provided electronically. In Poland, participants were required to provide hand signed declarations of consent via forms printed from the website and mailed to the local study team.

Participants were asked to provide information via a series of self-completed questionnaires using either a secure website or a telephone-based interactive voice recognition system (IVRS). Questionnaires were completed at study entry, over the duration of their pregnancy (completed every 2-4 weeks with the frequency decided by the participant at study entry) and shortly following the expected date of delivery (EDD) by website participants, or at study entry and shortly following the EDD only by IVRS participants. For pregnancies that ended prior to the EDD, participants reported they were no longer pregnant at their 2 or 4 weekly information request. Participants were also free to discontinue their participation at any stage, providing notification either by email or telephone.

For inclusion in the study participants were required to be currently pregnant, residing in one of four study countries, to have adequate natural language skills for that country, have internet or telephone access, and to be of legal age for the provision of their consent to participate (Denmark, Netherlands, and Poland – 18 years; United Kingdom – 16 years). In Denmark there was an additional requirement for participants to provide their civil registration number.

### Study Sample Size

Because this was an exploratory pilot study, an arbitrary target sample size of 4800 women (1200 per study location) was selected with the goal of recruiting participants over a 104-week period. However, due to time delays in obtaining the necessary approvals the recruitment period was reduced to 70 weeks.

### Study Recruitment and Data Collection

Participants were recruited from October 1, 2012 (recruitment week 1) until January 31, 2014 (recruitment week 70) in Denmark, the Netherlands, and the United Kingdom. Due to difficulties in arranging ethical approvals, the start date in Poland was further delayed until May 20, 2013 (recruitment week 34). Follow-up data, which are not further considered in this manuscript were collected from all participants until March 28, 2014. Participants received no incentives for enrolment or retention.

### Recruitment Strategies

A key recruitment objective was to use direct-to-participant advertisements wherever possible to recruit women early in their pregnancy, and at low or no cost. In the first 18 weeks of the recruitment period, low/no-cost advertisement methods were used in Denmark, the Netherlands, and the United Kingdom. These included posting promotional discussion topics in pregnancy online forums (United Kingdom – no cost), placing small hyperlinks on pregnancy-related websites (Netherlands – no cost), displaying leaflets and posters in community pharmacies and/or obstetric/midwifery units (Netherlands and United Kingdom – printing and delivery costs), communication through a social media profile (Facebook United Kingdom – no cost), and banner advertising in a pregnancy-specific section of a popular health and wellbeing website (Denmark – low cost). Subsequently, additional funding was provided for higher cost advertising methods, which included large digital banners (Denmark), hyperlinks (Netherlands), and small picture/text graphics (United Kingdom) placed on prominent pregnancy specific websites, adverts in emails sent to registered users of widely used pregnancy/health information, and parenting websites (all locations), an advert aired on regional/digital-Internet television channels (Poland), a full-page article in a regional newspaper and pregnancy magazine (Poland), and paid advertising on a social media site (Facebook – targeting British women aged 16-45 with interests in children, pregnancy, and health). Several examples of the adverts used to promote the study are provided in the supplementary appendix.

### Advertisement Method Cost-Effectiveness Analysis

To compare the international advertising costs, conversion of local currencies to Euros was performed using the exchange rate on March 10, 2014 (Euro:DKR 0.13, Euro:PZ 0.24, Euro:GBP 1.20, Euro:USD 0.72). Only advertising methods with associated costs (paid advertising methods) displayed via similar media platforms were grouped for comparison in this analysis. The cost-effectiveness of the various paid advertisement methods were compared as total cost per participant recruited (€ per participant).

### Stage of Pregnancy at Enrolment Analysis

All participants were requested to provide their EDD. Using this, the date of the last menstrual period (LMP; EDD-280 days) was calculated and the date of enrolment was defined as being within the first (1-90 days post-LMP) or second/third trimester (91-280+ days post-LMP). The proportion of participants in their first trimester at enrollment was compared between study locations and paid advertisement methods using Poisson regression (*P*<.05 indicating statistical significance). Statistical analysis was performed using Stata Version 13.1 [14].

## Results

### Participant Recruitment

In total the advertisements generated traffic of 43,220 unique subjects to the study website and 14 calls to the IVRS over the 70-week recruitment period. Of these women, 5.83% (2521/43,220) self-enrolled and began data entry. However, 455, including 13 of 14 who chose to provide data via the IVRS, did not complete the study entry questionnaire. As insufficient information was available from these participants, and only a single participant used the IVRS system, only the details of the 2065 active study participants who completed the study entry questionnaire via the website are discussed further (Denmark n=639, Netherlands n=476, Poland n=241, and the United Kingdom n=709).

**Figure 1:**
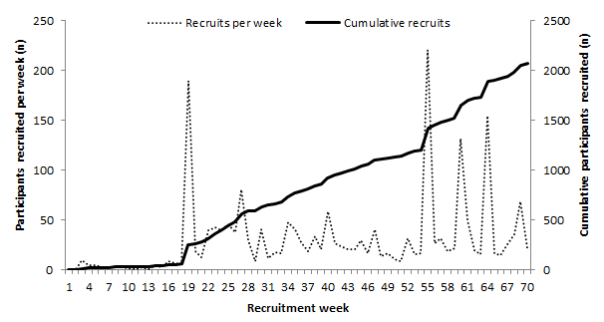
Timeline of participant recruitment (large scale email advertisements at weeks 19, 55, 60, and 64).

Figure 1 below describes the total number of active study participants joining the study in each week of the recruitment period. The low/no-cost advertisements used over the first 18 weeks of recruitment attracted a smaller than expected number of visitors to the website (n=1278), of which only 4% (52/1278) enrolled and provided study data. This prompted the use of higher cost advertisement methods, and Figure 1 clearly demonstrates the improvement in recruitment following their implementation (week 19+). 

Also, clearly seen from Figure 1 are the large increases in recruitment, which were observed during weeks 19, 55, 60, and 64, which were mainly produced by the large scale email advertisement campaigns where in excess of 100,000 emailed adverts were broadcast.

### Advertisement Method Cost-Effectiveness

Of the 2065 active study participants, 1867 were recruited through paid advertisements, which broadly fit into the following five categories: website advertisements, email broadcasts, leaflet promotions, television broadcasts, and social media advertisements. A description of the costs incurred for each of these categories, the number of participants recruited, and the resulting cost-per-participant is provided in Table 1. Where available we have also provided estimates of the total impact of the advertisements. For website/paid social media advertisements, we display the total number of advert impressions (ie, the number of times advertisement were displayed to users), for emails the total number broadcast, and for leaflets the total number printed.

**Table 1:** Number of active participants recruited by each of the paid advertisement methods.

Advertisement Methods		Denmark	Netherlands	Poland	United Kingdom	All locations
All Paid Methods						
	Total expenditure (€)	9912.00	19,010.00	10,843.00	16,962.00	56,727.00
	Participants recruited (n)	614	446	125	682	1867
	Cost per participant (€)	16.14	42.62	86.74	24.87	30.38
Website						
	Total expenditure (€)	9522.00	6002.00	276.00	576.00	16,376.00
	Total impressions (n)	2,124,341	1,789	183,652	567,556	2,877,338
	Participants recruited (n)	594	32	22	20	668
	Cost per participant (€)	16.03	187.56	12.55	28.80	24.52
Email						
	Total expenditure (€)	390.00	7412.00	5304.00	10,022.00	23,128.00
	Total emails sent (n)	14,000	80,214	94,500	120,442	309,156
	Participants recruited (n)	20	346	47	582	995
	Cost per participant (€)	19.50	21.42	112.85	17.22	23.24
Leaflet						
	Total expenditure (€)	-	5596.00	4355.00	6178.00	16,129.00
	Total leaflets printed (n)	-	15,030	19,850	13,250	47,880
	Participants recruited (n)	-	68	47	79	194
	Cost per participant (€)	-	82.29	92.66	78.20	83.14
Television						
	Total expenditure (€)	-	-	908.00	-	908.00
	Participants recruited (n)	-	-	9	-	9
	Cost per participant (€)	-	-	100.89	-	100.89
Paid Social Media						
	Total expenditure (€)	-	-	-	186.00	186.00
	Total impressions (n)	-	-	-	135,305	135,305
	Participants recruited (n)	-	-	-	1	1
	Cost per participant (€)	-	-	-	186.00	186.00

In total, €56,727 was spent on the paid advertising methods, which equated to €30.38 per active participant. In comparing the five broad categories of paid advertisements across all countries, the least cost-effective were social media (€186.00), television advertisements (€100.89), and leaflet advertising (€83.14), while website and email advertisements were the most cost-effective at €24.41 and €23.24, respectively. However, there were considerable international variations in the cost-effectiveness of the broad advertisement categories (Table 1). For example the cost-per-participant for website advertising in Denmark (€16.03), Poland (€12.54), and the United Kingdom (€28.80) was low in comparison with the Netherlands (€187.56), while for email advertising, costs in Poland were high (€112.85) in comparison with Denmark (€19.50), the Netherlands (€21.42), and the United Kingdom (€17.22). Leaflet advertising costs were found to be similar across the three study locations where they were used (Netherlands €82.29, Poland €92.66, United Kingdom €78.20).

### Recruitment of Participants in Early Pregnancy

Of the 2065 active participants recruited to the study 23.0% (475/2065) were in their first trimester at enrolment. Table 2 reports the number of active participants recruited by gestational age at enrolment stratified by study location. A Poisson regression comparing the proportion of first trimester enrolled participants across the locations identified a statistically significant difference (*P*<.001) driven by the high proportion of first trimester active participants recruited from Denmark (309/330, 48.4%) in comparison with the lower proportions recruited from the Netherlands (48/476, 10.1%), Poland (48/193, 19.9%), and the United Kingdom (70/709, 9.87%).

**Table 2:** Stage of pregnancy at which active participants were recruited to the study (SOP, stage of pregnancy, participants recruited in the second or third trimesters had reached at least 13-weeks’ gestation)

SOP		Denmark	Netherlands	Poland	United Kingdom	All Locations
		n (%)	n (%)	n (%)	n (%)	n (%)
First trimester		309 (48.4%)	48 (10.1%)	48 (19.9%)	70 (9.9%)	475 (23.0%)^a^
	≤4/40	58 (9.1%)	2 (0.4%)	3 (1.2%)	0 (0.0%)	63 (3.1%)
	5/40	53 (8.3%)	0 (0.0%)	4 (1.7%)	4 (0.6%)	61 (3.0%)
	6/40	53 (8.3%)	2 (0.4%)	5 (2.1%)	4 (0.6%)	64 (3.1%)
	7/40	37 (5.8%)	2 (0.4%)	5 (2.1%)	2 (0.3%)	46 (2.2%)
	8/40	29 (4.5%)	4 (0.8%)	8 (3.3%)	7 (1.0%)	48 (2.3%)
	9/40	23 (3.6%)	15 (3.2%)	0 (0.0%)	14 (2.0%)	52 (2.5%)
	10/40	21 (3.3%)	9 (1.9%)	4 (1.7%)	8 (1.1%)	42 (2.0%)
	11/40	20 (3.1%)	6 (1.3%)	9 (3.7%)	15 (2.1%)	50 (2.4%)
	12/40	15 (2.4%)	8 (1.7%)	10 (4.2%)	16 (2.3%)	49 (2.4%)
Second/third trimester		330 (51.6%)	428 (89.9%)	193 (80.1%)	639 (90.1%)	1590 (77.0%)
Total		639 (100.0%)	476 (100.0%)	241 (100.0%)	709 (100.0%)	2065 (100.0%)

^a^Poisson regression (*P*<.001) identifies a significant difference between the four study locations in the proportion of first trimester participants recruited driven by the high proportion of Danish participants recruited in the first trimester.

Table 3 compares the ability of the five broad categories of paid advertisement methods to recruit participants in their first trimester. A second Poisson regression, which compared the proportion of first trimester enrolled participants across three of five advertisement categories (television- and social media–recruited participants excluded due to small sample sizes) identified a statistically significant difference (*P*<.001) driven by the high proportion of total first trimester active participants recruited by website advertisements (317/668, 47.5%) in comparison with the email (72/995, 7.24%) and leaflet advertisements (53/194, 27.3%). Although website advertisements recruited the highest proportion of first trimester participants overall, as they did in Denmark, Poland, and the United Kingdom, in the Netherlands only 18.8% (6/32) of participants recruited via website adverts were in their first trimester compared with 41.2% (28/68) of leaflet recruited participants.

**Table 3:** Overview of the number of study participants recruited in the first trimester (T1) by each of the paid advertisement methods stratified by study location.

Advertisement Methods	Denmark	Netherlands	Poland	United Kingdom	Total
Total participants recruited by paid advertising *n*	614	446	125	682	1867
Total recruited in T1 n (% of total)	299 (48.7%)	47 (9.87%)	28 (22.4%)	68 (10.0%)	442 (23.7%)
Website n T1/total by method (%)	296/594 (49.8%)	6/32 (18.8%)	8/22 (36.4%)	7/20 (35.0%)	317/668 (47.5%)^a^
Email n T1/total by method (%)	3/20 (15.0%)	13/346 (3.8%)	11/47 (23.4%)	45/582 (7.7%)	72/995 (7.2%)
Leaflet n T1/total by method (%)	-	28/68 (41.2%)	9/47 (19.1%)	16/79 (20.3%)	53/194 (27.3%)
Television n T1/total by method (%)	-	-	0/9 (0.0%)	-	0/9 (0.0%)
Paid Social Median T1/total by method (%)	-	-	-	0/1 (0.0%)	0/1 (0.0%)

^a^Poisson regression (*P*<.001) identifies a significant difference in the proportion of first trimester study participants recruited between the website, email, and leaflet advertisement methods, driven by the high proportion of first trimester participants recruited by website advertisements.

## Discussion

### Principal Findings

In this non-interventional study of self-reported gestational exposures and pregnancy outcomes we found that low/no-cost advertisements were unsuitable for recruiting a high number of study participants in a short period of time. Higher-cost advertisements improved recruitment considerably although the costs were not insubstantial. Email and website advertisements performed preferentially to the other methods in terms of costs-per-participant recruited and overall website advertisements were substantially superior at recruiting participants in the first trimester. However, we did identify international variations in both the cost-effectiveness of the various advertisement methods used and in their ability to recruit participants in early pregnancy.

### Advertisement Cost-Effectiveness

We believe that our study represents the first attempt to assess the cost-effectiveness of direct-to-participant advertisement methods used in the recruitment of women to a pregnancy PV study. Available published data comparing the cost-effectiveness of different advertisement methods to recruit pregnant women to health research programs are limited [12,15,16]. One study showed advertising via social media (€25.00 per participant, based on 1,829,115 advert views over approximately 1 month, 624 clicks to the website, and 8 recruits) [15] to be more cost-effective than in our study (€186.00, based on 135,305 advert views over one month, 236 clicks to the website, and 1 recruit). Results from a second study were more consistent with our findings, suggesting that email advertising was the most cost-effective of the methods trialed [16], although in contrast to our findings website advertising was reported to be approximately three times less cost-effective than email advertising [16]. In addition, a final study identified a considerable reduction in total research costs, including recruitment costs, for studies conducted via the Internet in comparison with more traditional researcher interview techniques [12]. In this study, traditional researcher-led recruitment costs were approximated at €33 per participant, while Internet advertisement recruitment costs were lower than what we experienced overall (€24.52) at €11.68 per participant [12].

The key limitation of our cost-effectiveness analysis relates to the inability to account for any cumulative exposure to various advertisement materials. An additional limitation of the international comparisons specifically relates to the way in which we combined all the different advertisements used into five broad categories. For example, the website advertisements category combines cost-efficacy data from all the adverts placed in various locations on a variety of sites all with different levels of internet visibility (see Table 1). It is therefore plausible that the number of unique site visitors, which viewed the adverts varied between the websites that were grouped within this category. While it might have been expected that this would be controlled for by the cost of advertisement, with more prominent websites charging higher costs, we found this wasn’t comparable internationally. It was possible to advertise in prominent positions on popular Danish websites at a lower cost than that required for advertising on Dutch, Polish, or British websites with similar levels of web visibility.

### Recruitment of Participants in Early Pregnancy

One of the hypothesized advantages of collecting data from pregnant women recruited directly without the requirement for study promotion by a health care professional was that women could theoretically be recruited early in pregnancy, prior to seeking out any obstetric care. This was considered potentially beneficial in that it could provide surveillance over a gestational period not easily covered by traditional pregnancy PV/PE studies, collecting early pregnancy exposure and outcome data, which may otherwise be missed. Published data comparing the demographics of pregnant participants recruited through different advertisement methods are limited [17]. The single published study that we identified [17] did not investigate the differences in stage of pregnancy at enrollment for the various advertisement methods used. We therefore believe that our findings represent the first to demonstrate the variation in stage of pregnancy at enrollment by advertisement method.

It is likely that a main factor predicting an advertisement method’s ability to successfully recruit participants in early pregnancy is the time at which women naturally interact with the media platforms on which advertisements are displayed. For example, email advertisements were mainly sent to registered members of online pregnancy or parenting clubs; although women in early pregnancy are not prevented from registering with these, it is probable that most only do so in later stages of gestation (>90 days post LMP – outside the first trimester) when more confident that the pregnancy is likely to continue to term. In contrast, pregnancy/parenting information websites may be commonly viewed by women in the early stages of pregnancy investigating pregnancy symptoms or using due date calculators.

Overall the results of our study identified that website advertisements recruited a significantly higher proportion of women in early pregnancy in comparison with the alternative methods. The results also identified a significant difference in first trimester recruitment by study location, a finding that we believe was mainly influenced by the high proportion of Danish study participants which were recruited through website advertising (~93%).

In this pilot study, most women were recruited beyond the first trimester, but this is still of potential value for direct-to-participant pregnancy PV/PE studies [18]. For example details of early pregnancy maternal exposures and lifestyle choices may still be collected, albeit retrospectively, and therefore with a possible risk of introducing misclassification or recall biases.

### Future Research

For pregnancy PV studies specifically, the ability to detect increased incidences of rare gestational events such as specific congenital malformations among women taking a specific medication is dependent on the enrolment of a large study population. Prospective enrollment in early pregnancy is also considered advantageous as it is likely to minimize the introduction of some detrimental biases. Over the course of the PROTECT pregnancy study’s 70-week recruitment period, we conservatively estimate that more than 3 million pregnancies would have been recognized by women residing in the four study locations [19]. We therefore only enrolled a small percentage of the total pregnant population, which raises concerns regarding the ability of these methods to recruit a sufficiently large enough sample size to test associations between exposures and rare clinical events. In addition, the advertisement methods we employed were much more likely to recruit women in the later stages of pregnancy, which may introduce selection, recall, and misclassification biases. While we remain optimistic that study participant self-reporting of medication use and obstetric outcomes will prove advantageous for pregnancy PV/PE studies, future research is needed to identify advertisement strategies which are able to recruit large numbers of demographically representative pregnant women, preferably early in pregnancy and at a low cost. While the recruitment strategy employed in this study aimed to exclusively employ direct-to-participant advertisement methods, it is possible that recruitment may be improved using study promotion through clinicians such as general practitioners/family doctors who are often the first health professional to speak to patients about pregnancy.

### Conclusions

Of the various advertisement methods trialed in this pilot study, website and email advertisements were the most cost-effective, while website advertisements were the most suitable for enrolling participants in their first trimester. We believe these findings could prove useful for researchers looking to recruit a similar study cohort directly and without the intervention of health care professionals or academic researchers. However, our results also identify some concerns regarding the use of direct-to-participant advertisement methods as the sole strategy for recruiting women to a pregnancy PV study.

## Abbreviations

EDD: estimated due date
IVRS: interactive voice recognition system
LMP: last menstrual period
PE: pharmacoepidemiology
PV: pharmacovigilance
PROTECT: Pharmacoepidemiological Research on Outcomes of Therapeutics by a European Consortium
SOP: stage of pregnancy
